# Emerging role of mesenchymal stromal cells in gynecologic cancer therapy

**DOI:** 10.1186/s13287-023-03585-0

**Published:** 2023-12-05

**Authors:** Yizuo Song, Hejing Liu, Shuya Pan, Xinli Xiang, Miaomiao Ye, Xueqiong Zhu

**Affiliations:** Department of Obstetrics and Gynecology, The Second Affiliated Hospital and Yuying Children’s Hospital, Wenzhou Medical University, No. 109 Xueyuan Xi Road, Wenzhou, 325027 Zhejiang China

**Keywords:** Mesenchymal stromal cells, Stem cells, Tumor tropism, Therapy, Anti-tumorigenic, Pro-tumorigenic

## Abstract

Mesenchymal stromal cells (MSCs) show considerable promise in regenerative medicine with superior anti-fibrotic, immunomodulatory, and angiogenic functions. More recently, discovered with the tumor tropism, MSCs have been exploited as the basis of targeted cancer therapy. In this scenario, MSCs can directly home to tumor tissues and play anti-tumor properties. In addition, MSCs, MSC-derived exosomes and MSC-derived membranes are often developed as carriers for precisely delivering cytotoxic agents to cancer sites, including chemotherapeutic drugs, therapeutic genes, or oncolytic viruses. However, it has revealed the tumorigenic risk of MSCs as an important component within the tumor microenvironment, hampering the translation of MSC-based cancer therapies into clinical settings. Therefore, in this review, we introduce the specific tumor-tropic ability of MSCs and underlying mechanisms. We also summarize the current application of MSC-based therapeutic approaches in treating gynecologic cancers, mainly including cervical, ovarian, and endometrial cancers. Moreover, we discuss the main challenges that the current MSC-based cancer therapies are facing.

## Introduction

Mesenchymal stromal cells (MSCs) are a bulk population of multipotent stem cells, progenitors and differentiated cells [1, 2]. MSCs are first discovered within the bone marrow (namely bone marrow-derived MSC, BMSC) by Friedenstein and colleagues [3] in 1968. Since then, MSCs have been successfully isolated from other sources including dental pulp, adipose, skin, endometrium, umbilical cord, and placenta [4]. Owing to their low immunogenicity, superior anti-fibrotic and angiogenic roles, MSCs are considered as an excellent cell source for tissue regeneration in the last two decades [5]. In addition, other features of MSCs such as homing toward inflammatory or tumor sites have led to a rapid development in their therapeutic range beyond regenerative medicine [6, 7]. The stem cell population is a subgroup of progenitor cells with self-renewal and differentiated functionalities within the MSCs [8, 9]. Therefore, it is all of these cell populations, not just stem cell populations, that confer MSCs with notable secretory [10], immunomodulatory [11] and homing abilities [12].

Gynecologic cancers are still the prevalent and fatal diseases among women worldwide, mainly including cervical, ovarian, and endometrial cancers [13]. Surgical intervention, chemotherapy as well as radiotherapy play crucial roles in gynecologic cancer treatment [14–16]. However, the insufficient tumor selectivity, side effects to normal tissues and notorious resistance of anticancer drugs are the main obstacles to successful treatment of human malignancies [17]. The efficiency and safety of drug delivery have been improved to some extent through several nano-delivery methods based on the enhanced permeability and retention (EPR) effect [18]. However, the passive targeting effect is unable to achieve the sufficient accumulation of anticancer agents in tumor tissue regions. Therefore, more efficient drug delivery methods independent of the EPR effect are urgently needed.

Currently, cell-based therapies have gained increasingly clinical attraction in cancer treatment, such as MSCs [19, 20], red blood cells [21, 22], macrophages [23, 24], and T cells [25, 26]. Among these cell-based strategies, MSC-based cancer therapies have emerged with growing interests owing to the strong tumor-homing property of MSCs [27, 28]. Particularly, MSCs are reported to actively migrate to primary cancers or metastases after systemic infusion [29]. Several experimental evidence have shown the direct anticancer activities of MSCs [30–32]. Moreover, MSCs, MSC-derived exosomes and MSC-derived membranes (MSCMs) have been engineered to deliver chemotherapeutic drugs, therapeutic genes, and oncolytic viruses that precisely killing cancer cells [33–36]. However, it has been revealed that MSCs also play a tumorigenic function in cancer progression, limiting the translation of these therapies into clinical settings [37, 38].

Numerous studies that employ MSCs or MSCs’ derivatives for treating gynecologic cancers have been reported with mixed results over recent years [39–41], encouraging us to provide a comprehensive summary on this topic. In this review, we introduce the tumor tropism of MSCs and underlying mechanisms. Additionally, we summarize the current application of MSC-based therapeutic approaches in treating cervical, ovarian, and endometrial cancers. We also highlight the main challenges of MSC-based cancer therapies in gynecologic cancer treatment. Due to the space limitation, we sincerely apologize to those researchers for not citing their important works.

## Tumor tropism of MSCs

**Fig. 1:**
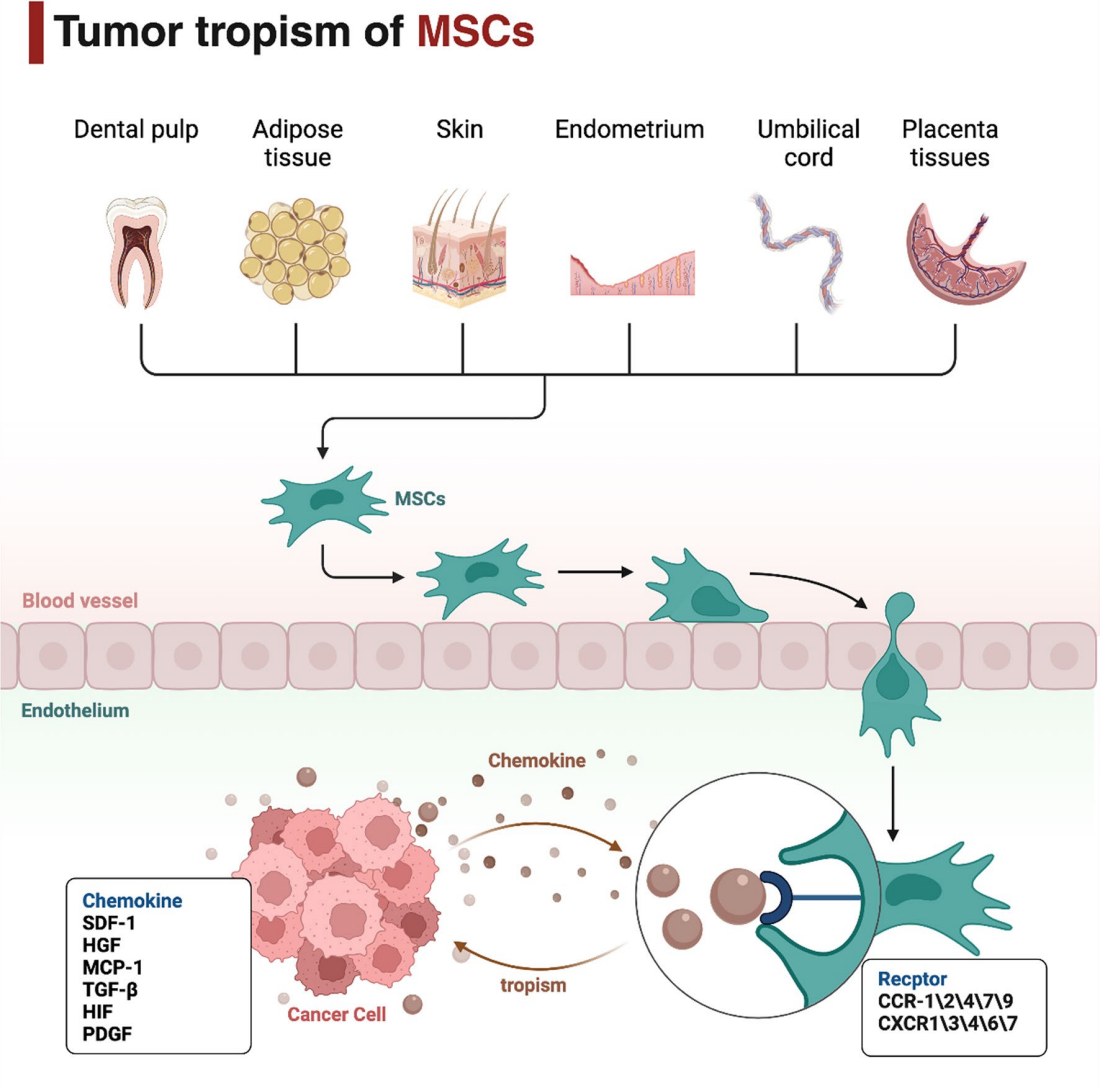
Schematic representation and the main mechanism involved in the tumor tropism of MSCs

The strong tropism toward cancer cells has predisposed MSCs to oncologic therapies in recent years [27, 28]. Importantly, MSCs are able to home toward injured or inflammatory tissues [42]. Thus, the tropism of MSCs to tumor sites is similar to the normal repair function in which the tumors are recognized by MSCs as “wounds that never heal” [43]. However, the mechanisms behind the tumor homing of MSCs are not fully understood. To the current knowledge, the tumor tropism of MSCs is attributed to the interaction of tumor cells-secreted chemokines and chemokine-associated receptors on the surface of MSCs [44]. First, the circulating MSCs in the blood stream are induced to roll and adhere into the endothelium by several chemokines and cell adhesion molecules expressed on the tumor blood vessels [45, 46]. Then, MSCs could migrate across the vessel wall into tumor sites upon the stimulation of chemokines from cancer cells [46, 47]. For instance, stromal cell-derived factor-1 (SDF-1), acting as an important ligand of CXCR4 and chemoattractant for MSCs, is constantly secreted by tumor cells [48, 49]. Reportedly, MSCs could express various types of chemokine receptors, including C–C chemokine receptor 1 (CCR1), CCR2, CCR4, CCR7, CCR9 and C-X-C chemokine receptor 1 (CXCR1), CXCR3, CXCR4, CXCR6 and CXCR7 [27, 45, 46]. The SDF-1/CXCR4 axis has been well investigated and displays a major role in inducing the tumor homing of MSCs [50]. In addition to the SDF-1/CXCR4 axis, multiple other molecules have been found to mediate the tumor tropism of MSCs, such as hepatocyte growth factor (HGF) [51], monocyte chemoattractant protein 1 (MCP-1) [52], tumor growth factor β (TGF-β) [53], hypoxia-inducible factor (HIF) [54], and platelet-derived growth factor (PDGF) [55]. This indicates that the molecular mechanisms whereby MSCs home to tumor cells need to be further elucidated. Based on the tumor tropism, more specific and effective therapeutic strategies using tropic MSCs have been developed for cancer therapy (Fig. 1).

## MSC-based therapies in gynecologic cancers

**Fig. 2:**
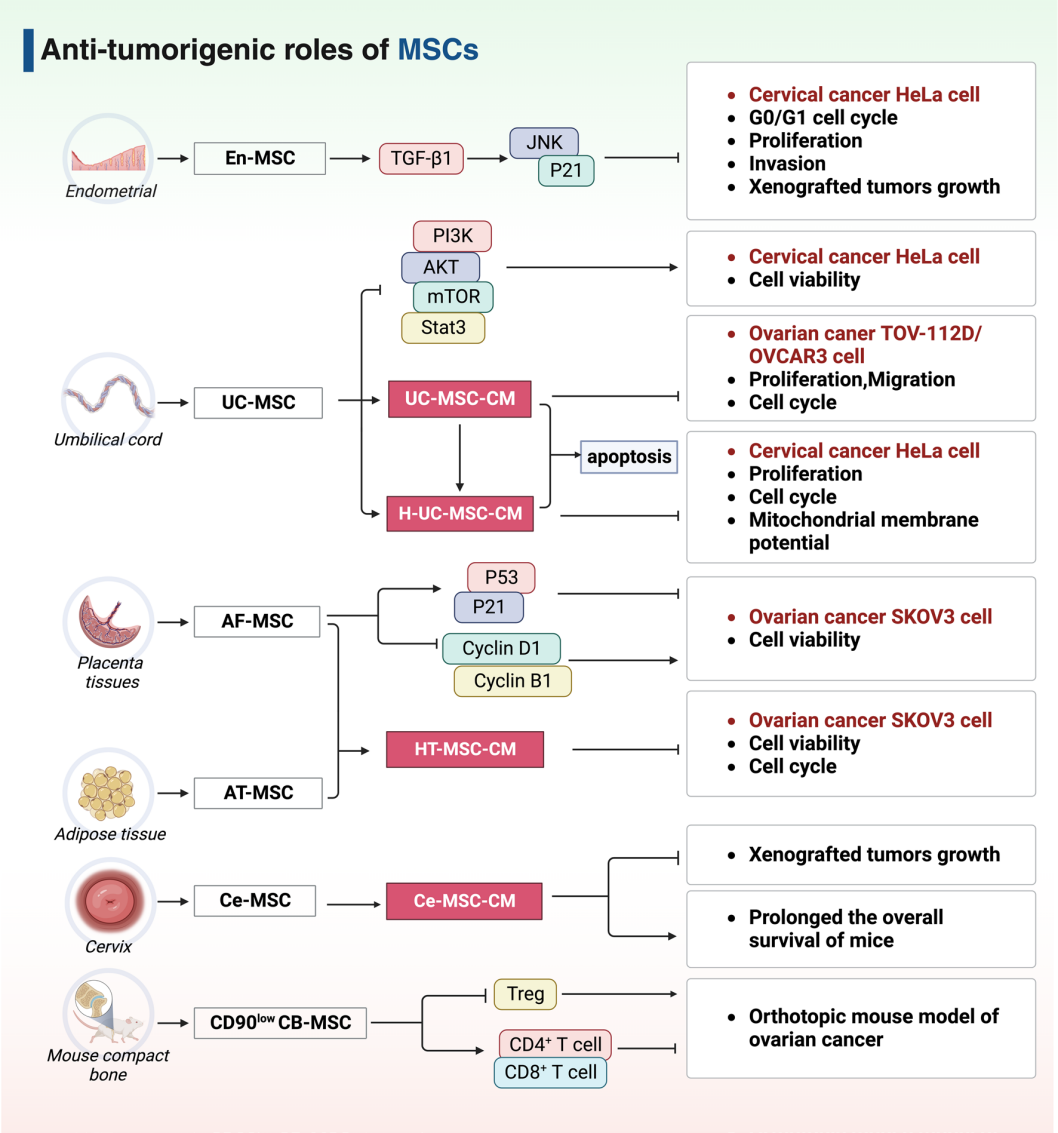
The major anti-tumorigenic functions of MSCs in gynecologic cancers

**Fig. 3:**
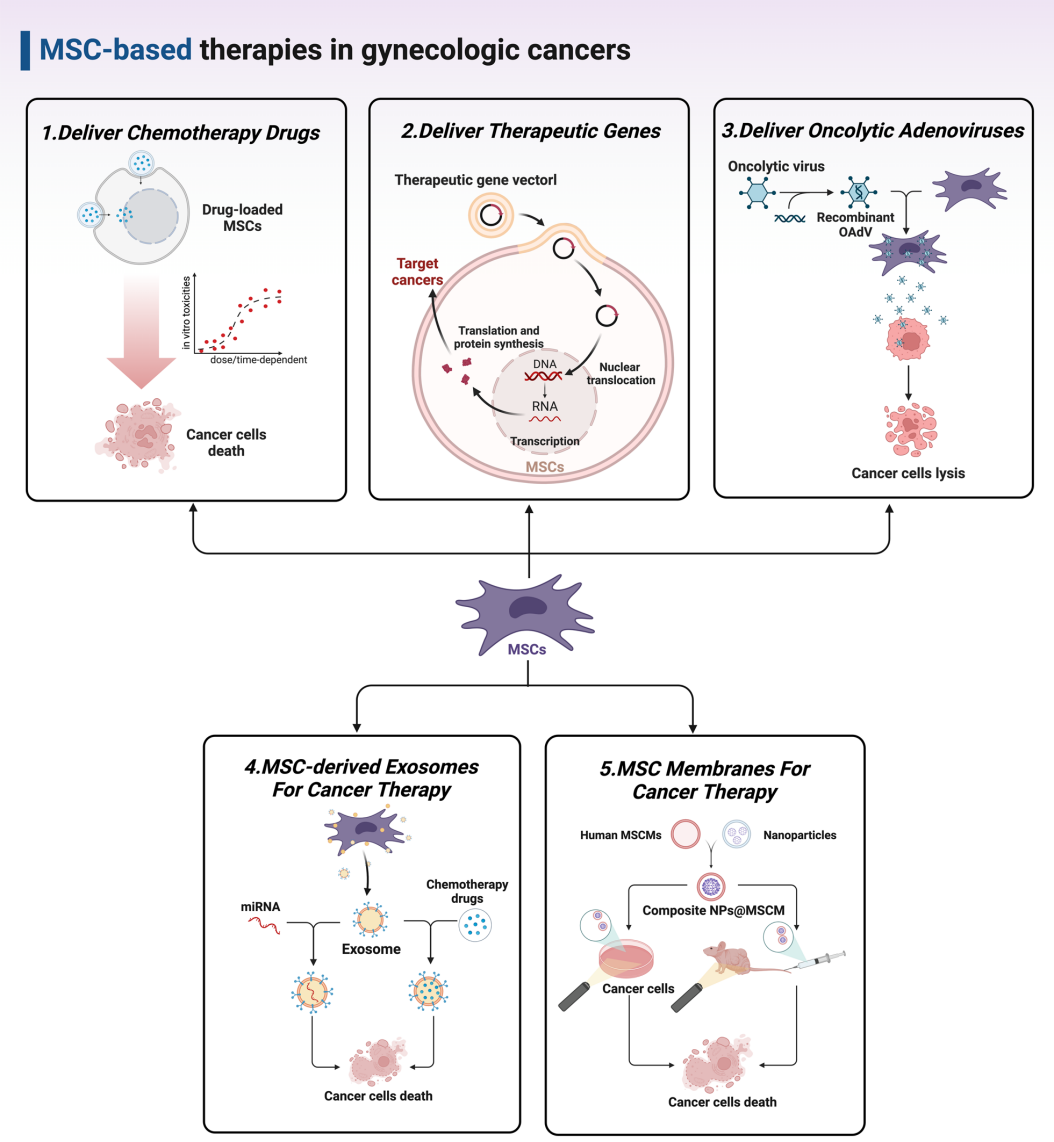
Schematic illustration of the current anticancer therapies based on engineered MSCs in gynecologic cancers

Numerous efforts have been made on developing MSC-based therapies for gynecologic cancer treatment. In the following section, we summarize therapeutic strategies involved in the use of MSCs, MSC-derived exosomes, and MSCMs as a powerful tool for killing cancer cells (Figs. 2 and 3).

### Anti-tumorigenic roles of MSCs

Despite few studies directly utilizing MSCs for tumor therapy, it is important to mention that tumor-suppressive roles of MSCs have been verified in several preclinical cervical and ovarian cancer models (Fig. 2).

#### Cervical cancer

The direct anticancer effects of MSCs have been studied on cervical cancer cells. For example, Liu et al. [32] isolated endometrial MSCs (En-MSC) from human menstrual blood and investigated their anticancer effects on cervical cancer cells. First, an *in vitro* transwell co-culture system was established by loading En-MSC and cervical cancer HeLa cells in the upper and lower chamber, respectively. And the co-culture results revealed that En-MSC blocked the cell cycle at G0/G1 phase, and suppressed the proliferation and invasion in HeLa cells. In vivo, a HeLa-bearing tumor xenograft mouse model was generated by subcutaneously co-injecting HeLa cells with En-MSCs at the density of 1:1. It was found that the average volume and weight of xenografted tumors were markedly decreased by En-MSC. Mechanistic experiments further revealed that En-MSC secreted TGF-β1 and activated the JNK/P21 signaling pathway in HeLa cells to exert anticancer effects. In another study, umbilical cord-derived MSC (UC-MSC) were co-cultured in direct contact with HeLa cells at a density ratio of 2:1. Experimental results found that UC-MSC could time-dependently inhibit the growth and invasion of HeLa cells through decreasing the activities of AKT/PI3K/STAT3/mTOR pathway and epithelial-mesenchymal transition (EMT), but did not alter the apoptosis and cell cycle [56].

Recently, therapies based on cellular secretome have emerged as promising treatments for human diseases [57]. Conditioned medium (CM) is one of the important secretome, which includes various bioactive factors that are secreted by MSCs or shed from their surface into the extracellular environment. One study had shown strong anticancer effects of CM derived from hypoxic UC-MSC (H-UC-MSC-CM) on cervical cancer HeLa cells. Specifically, H-UC-MSC-CM strongly decreased the viability and mitochondrial membrane potential, and induced the apoptosis and cell cycle arrest in HeLa cells [58].

#### Ovarian cancer

Aziz et al. [59] also employed the transwell co-culture system to explore the anticancer efforts of human amniotic fluid-derived MSC (AF-MSC) on ovarian cancer in vitro. It was reported that AF-MSC reduced the viability of ovarian cancer SKOV3 cells, which was related to increase in pro-apoptotic factors (p53 and p21) and decrease in anti-apoptotic factors (Cyclin D1 and B1).

Similarly, the CM from hyperthermia-treated (HT) adipose tissue-derived MSC (AT-MSC) and AF-MSC (HT-MSC-CM) also exerted tumor-suppressive effects on human ovarian cancer SKOV3 cells. The reduced viability and cell cycle arrest were observed after HS-MSC-CM treatment in SKOV3 cells by the morphologic, cell proliferation and Sub-G1 analyses [60]. Gauthaman and colleagues [61, 62] collected the UC-MSC-CM to treat ovarian cancer TOV-112D and OVCAR3 cell lines in vitro. Results demonstrated that the cell growth and migration were significantly inhibited by UC-MSC-CM, while the apoptosis and cell cycle arrest were induced in both cell lines [61, 62]. Using a tumor-bearing mouse model established by intraperitoneal injection of SKOV3 cells, Sendon-Lago et al. [63] found that intraperitoneal injection of human cervical MSC (Ce-MSC)-derived CM (Ce-MSC-CM) suppressed the tumor growth in vivo and prolonged the overall survival of mice. Moreover, Zeng et al. [64] evaluated the anti-tumor functions of MSC derived from mouse compact bone (CB-MSC) with low CD90 expression in a syngeneic orthotopic ovarian cancer model. Administration with CD90^low^ CB-MSC significantly suppressed tumor growth and increase the survival time of mice, and the anticancer effects were further enhanced after the combination of immune activator VIC-008. Notably, this was mediated by the activation of anti-tumoral CD4^+^ and CD8^+^ T cells and the reduction of Treg cells within the tumor microenvironment (TME). Collectively, these studies demonstrate the direct anticancer functions of MSCs in gynecologic cancers, which are partially through the paracrine signaling mechanisms.

### MSCs as delivery vehicles for anti-cancer agents

Mounting data have developed MSCs as vehicles for delivering therapeutic agents in a targeted way, including chemotherapy drugs, tumor-suppressive genes, and oncolytic viruses. Therefore, we will describe experimental evidence to summarize the applications of these engineered MSCs in gynecologic cancer therapy (Fig. 3).

#### Chemotherapy drugs

Poor availability into tumors and catastrophic side effects are major challenges that limit the efficacy of conventional chemotherapy drugs. Owing to the tumor-homing property, approaches that use MSCs have been designed to specifically enhance drug delivery and improve the therapeutic efficacy. To solve this issue, Sadhukha et al. [65] prepared a type of nano-engineered MSCs by incubating human MSCs with paclitaxel (PTX)-loaded poly(lactic-co-glycolic acid) (PLGA) nanoparticles (PTX-PLGA). Importantly, MSCs are highly resistant to drugs due to the upregulation of efflux transporters such as P-glycoprotein [66]. Hence, the incorporating PTX-PLGA nanoparticles did not influence the viability, differentiation and migratory capacities of MSCs. Moreover, a transwell system-based co-culture with nano-engineered MSCs generated dose- and time-dependent *in vitro* toxicities in human ovarian cystadenocarcinoma MA148 cells and lung cancer A549 cells. Although the in vivo anticancer effects of nano-engineered MSCs on ovarian cancer were not investigated, this study revealed that nano-engineered MSCs were selectively accumulated and retained in the orthotopic lung tumors.

Another study by Borghese et al. [67] determined the acquired anticancer activity of AT-MSC after priming with PTX (PTX-AT-MSC) via their ability to uptake drugs and release them into CM. Results found that priming with PTX slightly inhibited the capacities of AT-MSC to proliferate or migrate to ovarian cancer SKOV3 cells. Moreover, PTX-AT-MSC significantly inhibited the cell viability of SKOV3 cells in both two-dimensional (2D) models and three-dimensional (3D) heterospheroids. The cytotoxic effects between free PTX and PTX-AT-MSC-CM with equivalent amounts of PTX released by PTX-AT-MSC were also compared. Strikingly, PTX-AT-MSC-CM killed ovarian cancer A2780, SKOV3, and OVCAR3 cells, which was more active than free PTX. These data indicate the feasibility to engineer MSCs through chemotherapy drugs loading for reducing side effects and effectively treating ovarian cancer. Such more studies should be carried out for developing MSC-based drug delivery strategies in treating cervical and endometrial cancers.

#### Therapeutic genes

Despite few studies on the direct delivery of chemotherapy drugs by MSCs, researches using MSCs as vectors for gene therapy in treating gynecologic cancers are widely conducted. Herpes simplex virus thymidine kinase (HSVtk) is known as one of the most frequently used suicide gene therapy tools. Indeed, HSVtk could convert non-toxic ganciclovir (GCV) monophosphate or 5-fluorocytosine (5-FC) into toxic GCV or 5-fluorouracil (5-FU) to disrupt DNA synthesis [68]. In studies by Kenarkoohi et al. [69, 70], HSVtk-expressing mouse AT-MSC (AT-MSC^HSVtk^) was constructed by transduction with the lentiviral vector carrying *HSVtk* gene. It is noted that TC-1 cell lines, derived from primary lung epithelial cells of C57BL/6 mice and immortalized with human papilloma virus (HPV)-16 *E6/E7* oncogenes, are often used to establish cervical cancer mouse model [71]. After the formation of tumors, mice were intratumorally injected with AT-MSC^HSVtk^, followed by intraperitoneally administration of GCV after 14 days. Results demonstrated that AT-MSC^HSVtk^/GCV led to a significant decrease in tumor size in a common TC-1 cells-grafted cervical cancer model, which might be mediated by enhanced anticancer activities of natural killer (NK) cells and cytotoxic T cells. The inhibitory effects of AT-MSC^HSVtk^ were also investigated in highly metastatic ovarian cancer models. Toro et al. [72] subcutaneously co-injected ovarian cancer A2780 cells with AT-MSC^HSVtk^ in female nude mice, following by intraperitoneally injection of 5-FC after 14 days. A significantly decreased tumor volumes and prolonged survival time were observed in A2780 tumor-bearing mice after AT-MSC^HSVtk^/5-FC treatment.

Tumor necrosis factor (TNF)-related apoptosis-inducing ligand (TRAIL) is a transmembrane pro-apoptotic ligand that causes apoptosis of various cancer cells [73]. Although the anticancer activity of recombinant human TRAIL has been documented in several studies, in vivo use of recombinant TRAIL has a rapid renal clearance and short half-life [74]. Fortunately, this limitation can be overcome by engineering MSCs to stably produce and deliver TRAIL. In previous studies, TRAIL-armed BMSC (BMSC^TRAIL^) and AT-MSC (AT-MSC^TRAIL^) were successfully generated by retrovirus- and lentivirus-mediated transductions, respectively [36, 75]. Furthermore, BMSC^TRAIL^ and AT-MSC^TRAIL^ could target HeLa cells and caused a cell ratio-dependent apoptosis in vitro. In vivo studies in HeLa-bearing nude mice found that AT-MSC^TRAIL^ localized into tumors via either intravenous or subcutaneous administration and induced apoptosis without apparent toxicities to normal tissues [75].

Interferon-α (IFN-α), known as an important antiviral cytokine, plays also a tumor-suppressive role by inducing cell apoptosis [76]. Zhou et al. [77] genetically modified AF-MSC to overexpress IFN-α (AF-MSC^IFN−α^) and intravenously injected them into a mouse model with HeLa cell xenografts. The tumor tropism of AF-MSC^IFN−α^ was maintained and tumor growth was significantly restricted by AF-MSC^IFN−α^, which was mediated by cell proliferation suppression, angiogenesis inhibition, and apoptosis induction in cancer cells.

HPV E7 and E6 oncoproteins have emerged as specific immunotherapeutic targets in cervical cancer [78]. Previous studies show that E7 antigen-loaded dendritic cells (DCs), as classic antigen-presenting cells, elicits a tumor-specific T cell response in cervical cancer patients [79, 80]. Notably, MSCs could also act as antigen-presenting vehicles to develop anticancer immunotherapies. In the study by Bolhassani et al. [81], mouse BMSC was genetically modified to present E7 oncoprotein and small heat shock protein 27 (HSP27) (BMSC^E7+HSP27^). In addition, immunized with BMSC^E7+HSP27^-based vaccination significantly inhibited the tumor growth through boosting the E7-specific T-cell responses in TC-1-grafted cervical cancer mouse models. Taken together, these results provide evidence that MSCs could serve as good carriers for targeted gene transfer in treating cervical and ovarian cancers.

#### Oncolytic adenoviruses

Oncolytic viruses have emerged as promising therapeutics for treatment of human cancers in the last decade, whereby oncolytic viruses can be preferentially replicated within cancer cells and destroy them via direct lysis [82]. Among various oncolytic viruses, oncolytic adenovirus (OAdV) is the most commonly used viral platform [83]. However, the use of OAdV by direct injection is profoundly limited by nontarget infections and systemic toxicities [83].

Several studies have investigated the possibility of using MSCs as OAdV carriers owing to the documented tumor tropism. For instance, Komarova et al. [84] and Dembinski et al. [85] employed human BMSC to efficiently deliver Ad5/3 recombinant OAdV (Ad5/3-BMSC) or Ad5-Δ-24-RGD (D24RGD) recombinant OAdV (D24RGD-BMSC), respectively. Ad5/3-BMSC induced a significant death of SKOV3 cells after a direct contact co-culture, while D24RGD-BMSC displayed cytopathic effects on SKOV3 and OVCAR3 cells by the action of released D24RGD from supernatant. Furthermore, an orthotopic ovarian cancer animal model was established in these two studies by intraperitoneally injecting SKOV3 cells into female CB17 severe combined immunodeficient mice. By using this model, the tumor-homing capacities of Ad5/3-BMSC and D24RGD-BMSC were verified and the anticancer effects were evaluated. Specifically, BMSC-based delivery of Ad5/3 or D24RGD remarkably suppressed tumor growth in mice without systemic toxicity and prolonged the survival time, as compared to direct injection of free OAdV.

Another study by Alfano et al. [86] prepared a novel OAdV AR2011-expressing En-MSC (AR2011-En-MSC) to target ovarian cancer. Experimental results reported clear lytic activities of AR2011-En-MSC in vitro in both SKOV3 cells and malignant cells from ascitic fluids of ovarian cancer patients. Importantly, the antibodies-induced neutralization of AR2011 lytic effect in ascitic fluids was overridden by the good protection of En-MSC. Moreover, AR2011-En-MSC treatment was able to suppress tumor growth in nude mice carrying peritoneal malignant ovarian tumors. Hence, based on the intrinsic tumor-homing abilities, it provides a good delivery platform for OAdV to exert safer and stronger anti-tumor functions by riding on MSCs. However, studies using MSCs to deliver oncolytic viruses for treating cervical or endometrial cancers are still lacking.

### MSC-derived exosomes for cancer therapy

Exosomes are an important type of extracellular vesicles (EVs) secreted by living cells and mediate the cell-to-cell communication. Exosomes often carry specific bioactive cargoes like proteins, lipids, and nucleic acids to play various biological functions [87]. MSC-derived exosomes have been engineered as delivery vehicles which transfer tumor-suppressive microRNAs (miRNAs) to cervical or endometrial cancer cells for killing, including miR-302a [88], miR-144-3p [89], and miR-375 [90].

#### Cervical cancer

Two important oncogenic markers for cervical cancer were identified in another two studies, namely the centrosomal protein, 55 Kd (CEP55) [89] and maternal embryonic leucine zipper kinase (MELK) [90]. Intriguingly, previous bioinformatic analyses have proved that miR-144-3p and miR-375 are putative upstream negative regulators for CEP55 and MELK, respectively. In studies by Meng et al. [89] and Ding et al. [90], miR-144-3p- and miR-375-riched exosomes were extracted from human BMSC that was pre-treated by lentivirus transduction. Furthermore, the BMSC-derived exosomes could efficiently carry miR-144-3p or miR-375 into cervical cancer SiHa or C33A cells, playing significant inhibitory functions in the cell proliferation, invasion and migration via targeting CEP55 or MELK, respectively. The anticancer effects of miR-144-3p- or miR-375-contained exosomes were further confirmed in vivo through either intra-tumoral or intravenous injection in cervical cancer xenograft mouse models.

The exosomes released from MSCs have also been revealed to possess great therapeutic effects in cervical cancer via the delivery of chemotherapy drugs. In a most recent study, PTX was loaded into the exosomes derived from human UC-MSC (PTX-UC-MSC) by electroporation and then incubated with HeLa cells in vitro [91]. The PTX-UC-MSC-derived exosomes had good uptake in HeLa cells and significantly induced the apoptosis, and inhibited the proliferation and EMT in a dose-dependent manner.

#### Endometrial cancer

Li et al. [88] demonstrated that miR-302a was enriched in the exosomes derived from the established miR-302a-overexpressing human UC-MSC with lentivirus transduction. Moreover, the miR-302a-riched exosomes can be well internalized into endometrial cancer Ishikawa and ECC-1 cells, significantly inhibiting the cell proliferation and migration in vitro. The inhibitory effects induced by miR-302a-riched exosomes were consistent to that produced by direct overexpression of miR-302a in both Ishikawa and ECC-1 cells. The above results suggest that MSC-released exosomes can be used to efficiently treat cervical and endometrial cancers via targeted delivery of anticancer miRNAs or drugs.

### MSC membranes for cancer therapy

It should be noted that roles of MSCMs for drug encapsulation in the treatment of gynecologic cancers have not been fully studied. To date, there have been only one study reported in using MSCM-camouflaged nanosystems to treat cervical cancer [41]. The upconversion nanoparticles-based photodynamic therapy (PDT) agents are effective for cancer therapy, but limited by short time existing in the blood circulation and poor tumor-targeting ability. Modification by MSCMs on the surface of upconversion nanoparticles can overcome these limitations owing to the good tumor tropism of MSCs [92]. Hence, in the study by Gao et al. [41], a biomimetic PDT platform was prepared by fusing mesoporous silica-encapsulated β-NaYF4:Yb^3+^,Er^3+^ upconversion nanoparticles (UCNPs@SiO_2_) with human BMSCMs (SUCNPs@mSiO_2_). Experimental studies showed that SUCNPs@mSiO_2_ displayed MSC-mimicking long blood circulation time and tumor-targeting property with large accumulation at the tumor site. Further PDT experiments were performed under irradiation with 980 nm laser at 0.35 W/cm^2^ in HeLa cells and HeLa-bearing nude mouse models. In vitro co-culture or tail intravenous administration of SUCNPs@mSiO_2_ showed stronger suppressive efficacy in tumor growth than pure UCNPs@SiO_2_, providing the evidence that artificial modification of anticancer agents with natural MSCMs holds considerable potential for cervical cancer treatment by systemic administration. More studies using the biomimetic platform to treat ovarian and endometrial cancers should be carried out in the future.

## Challenges of MSC-based strategies in gynecologic cancer therapy

**Fig. 4:**
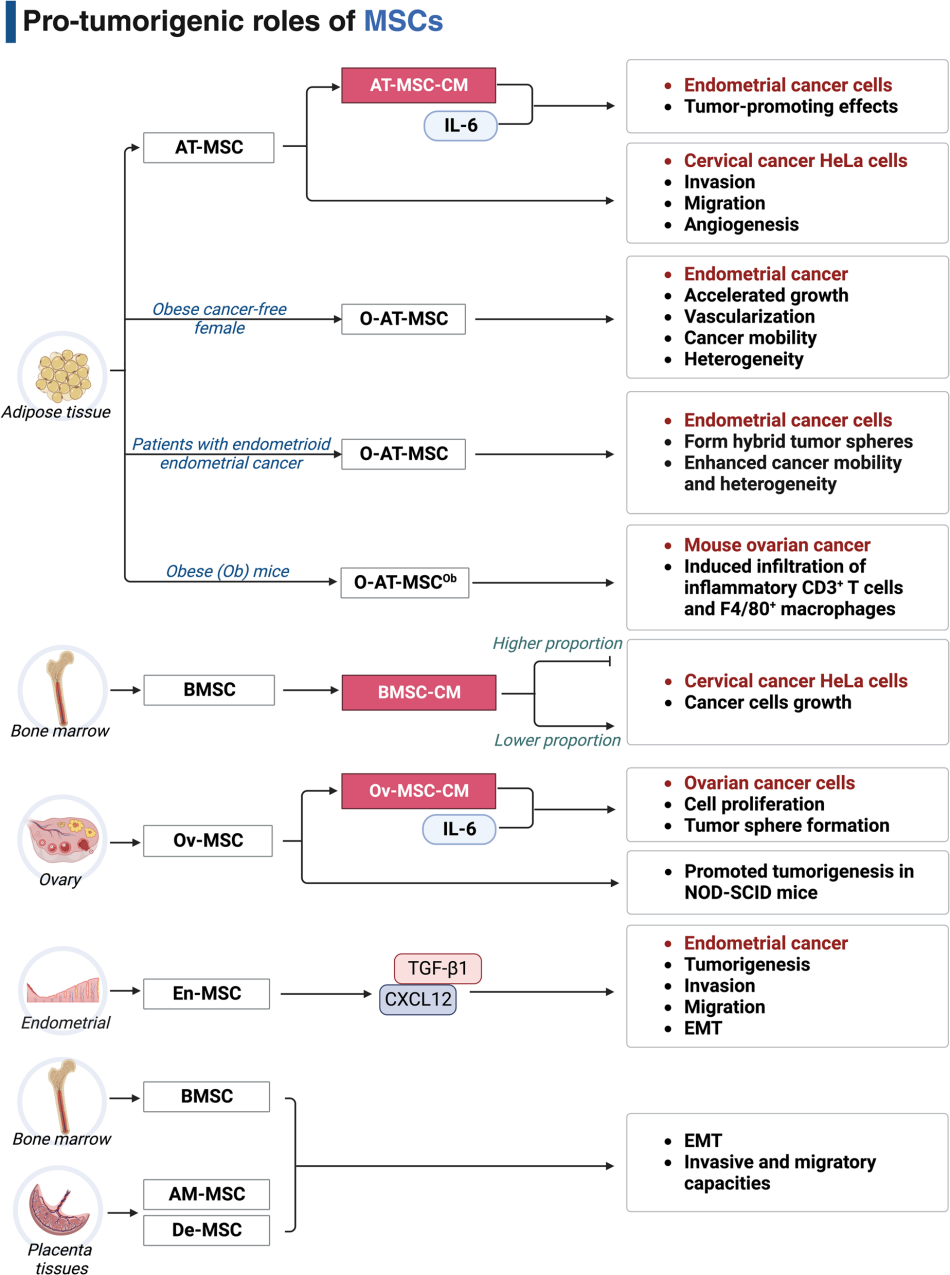
The pro-tumorigenic functions of MSCs in gynecologic cancers

Although the anticancer effects of MSCs have been well documented, MSC-based therapies are also challenged by oncogenic functions of MSCs as an exogenous regulators of cancer cells. In addition, although MSCs derived from distinct tissues have similar morphology and immunophenotypes, the homing ability of diverse MSCs may be discrepant and tissue-specific [93–95]. Therefore, we will discuss the tumorigenic roles of exogenous MSCs in gynecologic cancers below (Fig. 4). We will also highlight the tropic difference among various types of MSCs in cancer therapy.

### Pro-tumorigenic roles of MSCs

The pro-tumorigenic roles of exogenous MSCs that do not initially exist within TME have been described in some co-culture studies.

#### Cervical cancer

For example, Castro-Oropeza et al. [96] performed cellular experiments and bioinformatic analysis to demonstrate that human AT-MSC provoked increases in the invasion, migration, and angiogenesis of cervical cancer HeLa cells, which may be partially due to the occurrence of EMT during a transwell system-based co-culture. Moreover, interesting results were found by a previous study [97]. It was found that human BMSC exhibited both promotive and suppressive roles on the growth of HeLa cells under the CM, indirect transwell co-culture, and cell-to-cell contact co-culture models. Notably, the HeLa cells growth was increased by treatment of lower proportion of BMSC while decreased following the co-culture with higher proportion of BMSC. Therefore, we propose that the tumorigenic roles of MSCs may partly depend on the cell density administrated.

#### Ovarian cancer and endometrial cancer

Researchers have found that AT-MSC in adipose tissue can engraft into neighboring tumors to form supportive tumor stroma. Zhang et al. [98] isolated the AT-MSC from subcutaneous (SC-AT-MSC) and omental (O-AT-MSC) adipose tissues of lean (Le) and obese (Ob) mice. Despite similar stemness properties among these different AT-MSCs, O-AT-MSC^Ob^, but not SC-AT-MSC^Ob^, SC-AT-MSC^Le^, or O-AT-MSC^Le^, induced more infiltration of inflammatory CD3^+^ T cells and F4/80^+^ macrophages to support tumor growth after intraperitoneal co-injection with mouse ovarian cancer ID8 cells in C57BL/6 mice.

Klopp et al. [99] collected O-AT-MSC from an obese cancer-free female donor and compared its effects on endometrial cancer progression with SC-AT-MSC. By injecting different MSCs into the lower flank of NU/NU-foxn1^nu^ nude mice bearing HEC-1-A xenografts in the upper flank, O-AT-MSC was found to be more efficiently recruited to the tumors. Moreover, O-AT-MSC produced an accelerated growth and vascularization of endometrial tumors while SC-AT-MSC did not. In addition, Li et al. [100] first isolated O-AT-MSC from three patients diagnosed with endometrioid endometrial cancer at stages Ia to Ib. Direct co-cultured experiments found that O-AT-MSC could fuse with endometrial cancer Ishikawa and HEC-1-A cells to spontaneously form hybrid tumor spheres, leading to the enhanced cancer mobility and heterogeneity. According to body mass index (BMI), Li et al. [101] further obtained the O-AT-MSC from endometrial cancer patients with different BMI (BMI ≥ 30 and 18.5 < BMI < 24.9). Although the promotive effects of the O-AT-MSC on the proliferation and migration of HEC-1-A and Ishikawa cells were comparable, no significant differences were observed between the two groups. These data indicate MSCs that normally reside in different human tissues can be specifically recruited into tumor sites and are responsible for tumor growth. However, the tumor growth-promotive effects may be dependent on different tissue origins, different donors or even donors with different physical conditions.

#### Paracrine mechanism

Exogenous MSCs can also aggravate malignant traits of gynecologic cancer cells through contact-independent or contact-dependent paracrine mechanisms. For contact-independent way, Ding et al. [102] collected the ovarian MSC (Ov-MSC)-derived CM with abundant IL-6 and co-culture with ovarian cancer SKOV3 cells. The use of the IL-6-enriched Ov-MSC-CM promoted cell proliferation and tumor sphere formation. Furthermore, intraperitoneally co-injection of Ov-MSC with SKOV3 cells promoted tumorigenesis in NOD-SCID mice, which was blocked by IL-6 receptor antibody. Along similar lines, the En-MSC could indirectly interact with endometrial cancer cells (RL95-2, HEC-1-A, and Ishikawa), promoting both En-MSC and cancer cells to reciprocally secret TGF-β1 and CXCL12. The mutual interaction between TGF-β1 and CXCL12 led to the increased tumorigenesis, invasion, migration, and EMT of endometrial cancer cells in vitro and in vivo, which can be reversed by neutralizing antibodies of either CXCL12 or CXCR4 [103]. Notably, the same tumor-promoting effects were observed in using the IL-6-abundant CM from AT-MSC to co-culture Ishikawa cells in vitro and in vivo [104]. For contact-dependent way, So et al. [105] constructed a direct contact co-culture platform using various types of human MSCs (BMSC, amniotic membrane-derived MSC (AM-MSC), and decidua MSC (De-MSC)) and gynecologic cancer cells (SKOV3, IGROV-1; Ishikawa). Elevated IL-6 levels during direct co-culture in all groups significantly promoted the EMT of cancer cells, as manifested by enhanced invasive and migratory capacities.

### Differences in tumor tropism

The exact mechanism underlying the cancer tropism of MSCs has not been fully elucidated. Transplanted MSCs with higher tumor tropism are more prone to reach lesion sites and exert more effective therapeutic functions [106, 107]. Since the chemokines-receptors axis plays a critical role in MSCs tumor homing, the different expression profiles of chemokines from cancer cells or chemokine receptors in various MSCs may account for the tropism difference. Our group performed transwell experiments to compare the homing ability differences of four types of MSCs toward cervical cancer cells [108]. Results showed that chorionic plate-derived MSC (CP-MSC) displayed a stronger tropic property to SiHa and HeLa cells, as compared to AT-MSC, UC-MSC, and AM-MSC. Furthermore, a gradient descending expression of CXCR4 was observed in these different types of MSCs by western blotting analysis, with the highest level in CP-MSC. Therefore, a comprehensive screening of different homing abilities of each origin of MSCs to different cancer types should be exploited in the future. Based on the screening, selection of specific MSCs could permit more precise delivery of MSCs to cancer sites for therapeutic purposes.

## Conclusions and perspectives

The specific tumor-homing properties of MSCs have attracted increased attention by researchers to use MSCs to treat human gynecologic cancers. MSCs can possess the direct anti-tumoral functions toward cancer cells, but in most cases, they are functionally or genetically engineered to deliver chemotherapeutics, suicide genes, or oncolytic viruses in gynecologic cancer therapy. Furthermore, MSC-derived exosomes and MSCMs can be extracted as a bioactive carrier of anticancer drugs, miRNAs, or multifunctional composite nanoparticles. However, several questions or challenges need to be addressed before translating the MSC-based cancer therapies into clinical settings. First, MSCs possess pro-tumorigenic and anti-tumorigenic roles in cancer development. However, the reported discrepancies with respect to the capacity of MSCs to promote or inhibit tumorigenesis are based on the differences of experimental settings including animal models, cell lines, doses, and duration times of treatment. Therefore, whether MSCs should be considered as anticancer agents or a therapeutic target in cancer treatment is still a matter of debate. How to reduce the extrinsic tumorigenicity of exogenous MSCs such as functional modification will be another topic we need to focus on. Second, different tropism abilities require further delineation about the homing map of each type of MSCs in various cancers, which will guide the selection of proper MSCs in cancer therapy. Third, it is necessary to develop advanced extraction methods for scalably producing MSC-derived exosomes or MSCMs in the future. Lastly, up to date, there are only two clinical trials accessing the MSC-based therapies for the treatment of ovarian cancer that have been registered on *ClinicalTrials. Gov* database (NCT02530047 and NCT02068794). However, no data have been published from such trials. Moreover, the trials concerning the therapeutic value of MSCs on cervical or endometrial cancers are still lacking. By solving these challenges, we believe that MSC-based therapeutic strategies could more efficiently and safely kill cancer cells in human gynecologic cancers.

## Data Availability

Not applicable.

## Abbreviations

2D: Two-dimensional
3D: Three-dimensional
5-FC: 5-Fluorocytosine
5-FU: 5-Fluorouracil
AF-MSC: Amniotic fluid-derived MSC
AM-MSC: Amniotic membrane-derived MSC
AT-MSC: Adipose tissue-derived MSC
BMI: Body mass index
BMSC: Bone marrow-derived MSC
CB-MSC: Compact bone-derived MSC
CCR1: C–C Chemokine receptor 1
Ce-MSC: Cervical MSC
CEP55: Centrosomal protein, 55 Kd
CM: Conditioned medium
CP-MSC: Chorionic plate-derived MSC
CXCR1: C-X-C Chemokine receptor 1
DC: Dendritic cell
De-MSC: Decidua MSC
EmCa-MSC: Endometrial cancer-derived MSC
EMT: Epithelial-mesenchymal transition
En-MSC: Endometrial MSCs
EPR: Enhanced permeability and retention
EV: Extracellular vesicle
GCV: Ganciclovir
HGF: Hepatocyte growth factor
HIF: Hypoxia-inducible factor
HPV: Human papilloma virus
HSP27: Heat shock protein 27
HSVtk: Herpes simplex virus thymidine kinase
HT: Hyperthermia-treated
H-UC-MSC-CM: Hypoxic UC-MSC
IFN-α: Interferon-α
IGFBP-6: Insulin-like growth factor-binding protein 6
MCP-1: Monocyte chemoattractant protein 1
MELK: Maternal embryonic leucine zipper kinase
miRNA: MicroRNA
MSC: Mesenchymal stromal cell
MSCM: MSC-derived membrane
NK: Natural killer
OAdV: Oncolytic adenovirus
OvCa-MSC: Ovarian cancer-derived MSC
Ov-MSC: Ovarian MSC
PDGF: Platelet-derived growth factor
PDT: Photodynamic therapy
PLGA: Poly(lactic-co-glycolic acid)
PTX: Paclitaxel
SDF-1: Stromal cell-derived factor-1
TGF-β: Tumor growth factor β
TME: Tumor microenvironment
TNF: Tumor necrosis factor
TRAIL: Tumor necrosis factor related apoptosis-inducing ligand
UC-MSC: Umbilical cord-derived MSC
